# The Multi-State Epigenetic Pacemaker enables the identification of combinations of factors that influence DNA methylation

**DOI:** 10.1007/s11357-024-01414-7

**Published:** 2024-11-16

**Authors:** Colin Farrell, Keshiv Tandon, Roberto Ferrari, Kalsuda Lapborisuth, Rahil Modi, Sagi Snir, Matteo Pellegrini

**Affiliations:** 1 Dept. of Molecular, Cell and Developmental Biology, University of California, Los Angeles, 90095 CA USA; 2 Dept. of Chemistry, Life Sciences and Environmental Sustainability, Laboratory of Molecular Cell Biology of the Epigenome (MCBE), University of Parma, Parma, Italy; 3 Dept. of Evolutionary Biology, University of Haifa, Haifa, Israel

## Abstract

Epigenetic clocks, DNA methylation-based predictive models of chronological age, are often utilized to study aging associated biology. Despite their widespread use, these methods do not account for other factors that also contribute to the variability of DNA methylation data. For example, many CpG sites show strong sex-specific or cell-type-specific patterns that likely impact the predictions of epigenetic age. To overcome these limitations, we developed a multidimensional extension of the Epigenetic Pacemaker, the Multi-state Epigenetic Pacemaker (MSEPM). We show that the MSEPM is capable of accurately modeling multiple methylation-associated factors simultaneously, while also providing site-specific models that describe the per site relationship between methylation and these factors. We utilized the MSEPM with a large aggregate cohort of blood methylation data to construct models of the effects of age-, sex-, and cell-type heterogeneity on DNA methylation. We found that these models capture a large faction of the variability at thousands of DNA methylation sites. Moreover, this approach allows us to identify sites that are primarily affected by aging and no other factors. An analysis of these sites reveals that those that lose methylation over time are enriched for CTCF transcription factor chip peaks, while those that gain methylation over time are associated with bivalent promoters of genes that are not expressed in blood. These observations suggest mechanisms that underlie age-associated methylation changes and suggest that age-associated increases in methylation may not have strong functional consequences on cell states. In conclusion, the MSEPM is capable of accurately modeling multiple methylation-associated factors, and the models produced can illuminate site-specific combinations of factors that affect methylation dynamics.

**Supplementary Information:**

The online version contains supplementary material available at 10.1007/s11357-024-01414-7.

## Introduction

DNA methylation, the addition of a methyl group to the fifth carbon of the cytosine pyrimidine ring, is associated with the topological organization of the cellular genome, gene expression, and the state of a cell. Within a population of cells, the methylation pattern at certain sites can change predictably with the age of the individual from which the cells are drawn. This predictable nature of DNA methylation has led to the development of accurate DNA methylation-based predictive models for age and health, termed epigenetic clocks. The difference between the predicted and the expected epigenetic age given an individual’s chronological age has been interpreted as a measure of age acceleration [1] and has been associated with mortality [2, 3] and other adverse health outcomes [4–8].

However, epigenetic clocks suffer from several limitations that limit the interpretability of their predictions and the underlying mechanisms. Epigenetic clocks are generally trained by using penalized regression-based methods that attempt to minimize the difference between the predicted and observed value of age. As a result, as the error between predicted and observed age is decreased, the associations between age acceleration and mortality disappear [9]. Second-generation epigenetic clocks attempt to resolve this issue by fitting a measure of human health, rather than age, and as a result, these clocks are generally more sensitive to individual health status [10–12]. However, while the response variable is modified in these clocks, the method used to fit the clock is largely the same. Epigenetic clocks are generally trained using regularized regression models, where the likelihood is maximized by minimizing the difference between the observed and predicted response variable subject to the elastic net penalty, $\lambda _1$ and $\lambda _2$. Methylation sites that increase model error and are influenced by other relevant factors such as smoking or obesity may be discarded during model fitting, thus limiting the ability of this approach to account for the effects of these extraneous factors on epigenetic aging.

As an alternative to penalized regression-based methods, we previously developed an evolutionary-based model for epigenetic dynamics, the Epigenetic Pacemaker (EPM)[13, 14]. The EPM attempts to minimize the difference between observed and predicted methylation values amongst a collection of sites through the implementation of a conditional expectation maximization algorithm [15]. Under the EPM, the observed methylation status of a collection of sites is modeled linearly with respect to an input factor of interest, such as age. A hidden epigenetic state, that is related to the initial factor, but not necessarily linearly, is learned through the course of model fitting. The EPM can capture the non-linear relationship between methylation and age [16] and outputs an interpretable model for each site. However, both the EPM and regression-based methods suffer from the same limitation, which is that they are limited to a single trait predicted by, or used to model, observed methylation patterns. In reality, the observed methylation landscape is likely impacted by a variety of factors that act simultaneously to produce the observed methylome of an individual.

To overcome this limitation, we have developed a multidimensional extension of the EPM, the Multi-State Epigenetic Pacemaker (MSEPM). We show that the MSEPM can accurately model site-specific methylation variation driven by several factors and, given a trained model, accurately predict the values of the factors associated with an individual’s observed methylation profile in both simulated methylation datasets and a large aggregate blood tissue methylation dataset. Importantly, as factors that explain the observed methylation profile of an individual are added to the model, the ability to model the factors and methylation values improves. Additionally, we show that sites with similar associations to modeled factors cluster together and are enriched for specific transcription factors. Therefore, unlike traditional epigenetic clocks, the MSEPM allows us to study mechanisms that may underlie age-associated methylation changes. In our large dataset of blood samples, we find that sites that increase methylation with age are enriched for bivalent promoters and are proximal to genes that are lowly expressed in blood. These results suggest that positively age-associated sites may not have a significant functional impact on aging traits. The MSEPM is available as a Python package with scikit-learn style syntax under a MIT license at https://github.com/NuttyLogic/MultistateEpigeneticPacemaker.

## Methods

### Multi-State Epigenetic Pacemaker model

The MSEPM model describes the observed methylation at site *i* and for individual *j*, $\hat{m_{i,j}}$, as a weighted linear combination of *k* individual epigenetic factors $p_{k,j}$.

$$\hat{m_{i,j}} = r^0_i + \sum ^n_{k=1} p_{j,k} r_{i,k}$$

where *k* epigenetic factors are weighted by *k* site-specific epigenetic rates of change, $r_{i,k}$, and offset by a site-specific intercept term, $r^0_i$. Site parameters, $r_{i,k}$ and $r^0_i$, are characteristic of the site and shared amongst all individuals while epigenetic factors, $p_{j,k}$, are characteristic of an individual and are the same across all sites for that individual. In practice, the observed methylation value is also dependent on a normally distributed error term $\epsilon _{i,j}$.

$$\hat{m_{i,j}} = r^0_i + \sum ^n_{k=1} p_{j,k} r_{i,k} + \epsilon _{i,j}$$

Under this model, epigenetic factors are related to observable individual factors $p_{k,j}^0$, such as chronological age, sex, and cell types, but may be transformed relative to observable factors. The epigenetic age factor, for example, often has a non-linear relationship with the observed age [16]. The MSEPM learns the appropriate transformation during model fitting to describe the observed methylation status linearly in terms of the epigenetic age factor, but not linearly with age.

Given an input matrix $\hat{M} = [m_{i,j}]$ of methylation values for *i* sites and matched observable epigenetic factors $\hat{P^0} = [p_{j,k}^0]$ for *j* individuals, the objective of the MSEPM is to find the optimal values of $r_{i,k}$ and $p_{j,k}$ that minimize the residual sum of square (RSS) error,

$$\epsilon _{i,j}^2 = (m_{i,j} - r^0_{i} - \sum ^n_{k=1} p_{j,k} r_{i,k})^2$$

This is accomplished through the implementation of a conditional expectation maximization algorithm. The maximum likelihood (ML) values of $r_{i,k}$ and $r^0_i$ can be solved using ordinary least squares (OLS) regression. Provided the ML estimates for $r_{i,k}$, the site coefficients are fixed and epigenetic factors, $p_{j,k}$, are updated by minimizing the RSS across all *i* sites using gradient descent,

$$p_{j,k}^{n+1} = p_{j,k}^n - \lambda \nabla F(p_{j,k})$$

where $\lambda$ is a specified learning rate. The optimization is accomplished by alternating between optimizing $r_{i,K}$ and $p_{j,k}$ until the reduction in the sum of the site RSS is below a specified threshold or a set number of iterations is reached. Importantly, while the ML values of $p_{j,k}$ are by definition linear with the methylation status at any site, the original input factors for $p_{j,k}^0$ may not be.

Provided a trained MSEPM model and an unobserved methylation matrix, epigenetic factors are estimated by calculating each independent OLS for solution all *i* sites given the $r_{i,k}$ coefficients set for the respective input factor. These epigenetic factors can then be used to find the expected methylation value using the trained individual site models where

$$E[m_{i,j}] = r_{i,0} + P_{j} \dot{R}_{i}$$

where $P_{j} \dot{R}_{i}$ is a matrix of point values *p* and *r*.

### MSEPM simulation framework

We implemented a simulation framework using the MSEPM formulation to evaluate the performance of the MSEPM model under various conditions. To simulate the association between methylation status and an observable factor, we modeled the epigenetic factor $p_{k,j}$ as function of time, or age, and magnitude, $p_{k,j}^0$ with a non-linear transformation $\gamma _{k}$, where $p_{k,j} = Age_j p_{k,j}^{0,\gamma _{k}}$. In practice, the value of the $p_{k,j}$ is often unknown and the association between methylation status and $p_{k,j}$ is inferred through the observable factor $p_{k,j}^0$.

Methylation sites were simulated by first randomly setting the range of the methylation site, $-1< \delta < 1$ a site intercept, $r^0_i$, and the site error, $\sigma _i \sim \mathcal {U}(0.025, 0.05)$. The possible range of the methylation site is described by the initial methylation value, $m_0 ~ \beta (.2,.2)$, and the target methylation value, $m_t$, where the range is $\delta _i = m_t - m_o$. $\beta (.2,.2)$ is the beta distribution with its parameters for randomly setting the sample. The value of $m_t$ is set conditionally to ensure site variability is always larger than some specified threshold, $\theta$, where $\theta \le |\delta | \ge . r^0_i ~ \beta (.2,.2)$.

Simulated methylation sites are then randomly associated with a combination of zero, one, or multiple epigenetic factors. Rates for sites associated with multiple factors were set by sampling from a uniform distribution. The weighted factor rates are normalized, so the input combination of traits describes the range of the simulated site, $\delta$. If a site is associated with no factors, the observed methylation status of a site is described by a random normal with a characteristic offset, $\hat{m_i} = r_{0,i} + N(\mu , \sigma )$.

### Blood MSEPM model training

MSEPM models were trained using a large aggregate dataset of blood-derived methylation data from 17 publicly available Datasets [7, 17–32]. Illumina methylation 450K Beadchip methylation array IDAT files were processed using minfi [33] (v1.34.0). Sample IDAT files were processed in batches according to GEO series and Beadchip identification. Methylation values within each batch were normal-exponential normalized using out-of-band probes [34]. Blood cell-type counts were estimated using a regression calibration approach [35], and sex predictions were made using the median intensity measurements of the X and Y chromosomes as implemented in minfi [33]. Samples were filtered for quality control using the relative intensity of the methylated and unmethylated probes. Samples were used for downstream analysis if the sample median methylation probe intensity was greater than 10.5 and the difference between the observed and expected median unmethylation probe intensity is less than 0.4, where the expected median unmethylated intensity is described by $E[intensity_{unmethylated}] = 0.66 intensity_{methylated} + 3.718$. This resulted in a total of 5687 samples.

We trained MSEPM models using data assembled from four GEO series [20, 22, 29, 36] ($n=1605$). The samples were randomly split into training ($n=1203$) and validation ($n=402$) sets stratified by age. Methylation values for all samples were quantile normalized by probe type [37] using the median site methylation values across all training samples for each methylation site. Training set blood cell-type abundance estimates were used to train a principal component analysis (PCA) model which was then used to calculate cell type PCA estimates for the validation and testing sets. Methylation sites were selected for modeling with MSEPM if the site methylation values were correlated with age ($n=276$), sex ($n=49$), CT-PC1 ($n=120$), CT-PC2 ($n=116$), or a combination of factors ($n=238$) by absolute Pearson correlation coefficient, where an absolute Pearson correlation coefficient is greater than 0.7, 0.995, 0.92, and 0.64 for age, sex, CT-PC1, and CT-PC2, respectively. Sites with a sum of absolute Pearson coefficients across the four factors greater than 1.8 were also included $(n=238)$ for a total of 778 methylation sites. Min-max, (0–1), scalers were fit using the training input features. Validation and testing sample features were transformed with the trained scalers. Age was min-max scaled on a range from 0 to 100 years. MSEPM models were trained with a learning rate of 0.01 with an iteration limit of 200.

### Blood MSEPM model cluster transcription factor overlap analysis

We evaluated the relationship between modeled sites, input factors, and regulatory transcription factors using overlap enrichment analysis. We built a custom transcription factor reference set using ENCODE V4 transcription factor chromatin immunoprecipitation [38, 39] (release 1.4.0 - 2.1.2) irreproducible discovery rate narrow bed peaks, which contains peaks with high-rank consistency between replicates, that were not audited for non-compliance or errors. GRCh38 region coordinates were lifted to GRCh37 coordinates using liftOver [40]. The overlap reference contains 714 transcription factor targets from 1621 accession IDs.

We then performed hierarchical clustering of the four-factor MSEPM model sites based on the similarity of their regression coefficients. Individual methylation site coefficients were first normalized by the standard deviation of methylation values of the site among the training samples, $r_{i,k} / \sigma _{i}$. A distance matrix was then created by taking the Euclidean distance between the normalized site model coefficients. Sites were then clustered using Ward’s method which seeks to minimize within cluster variance by minimizing the increase in the error sum of squares (ESS) through successive cluster fusions. Clusters are labeled by tree cutting at a height of 18. All clustering analysis was carried out using SciPy v1.6.3 [41].

Transcription factor enrichment analysis was performed with LOLA [42] which assesses the genomic region set overlap between a set of query regions and a set of reference regions, within a specified shared background set, using Fisher’s exact test. Overlap analysis was performed for sites within a cluster against the ENCODE V4 reference region (1BP minimum overlap) using all sites assayed with Infinium HumanMethylation450K BeadChip as background.

### Clustering sites with age-associated increases in methylation

To better understand age-associated methylation in whole blood, we examined each site within MSEPM four-factor blood model cluster 7 individually, as this cluster contains sites that have methylation that increases with age but is not strongly affected by other factors. Using the EWAS Data Hub (Xiong, et al. 2016), we validated our results by obtaining additional methylation by age data in whole blood for each site in the cluster (McCartney, et al. 2019). We created a matrix with every sample and its associated methylation and age from cluster 7 and then used age-associated methylation levels to create a clustered heatmap using the Matlab function Clustergram. We then clustered the tree into four groups which were analyzed separately.

We also identified the genes that were proximal to each site using Cistrome-GO (Li et al. 2019). We then examined the expression of the genes across tissues in the Genotype-Tissue Expression (GTEx) database database. We used the GTEx Multi Gene Query to find which tissues those genes belonged to.

We utilized the Toolkit for Cistrome Data Browser [43, 44] for the analysis of significant factors in each cluster. This allowed us to input .bed files of each sub-cluster and generate a GIGGLE score for specific transcription factors, histone marks, and chromatin regions to assess the significance of these elements. A GIGGLE score tailored ranking of loci based on the overlap of genomic features provided by the user [45].

### H3K4me3 enrichment analysis

Enrichment of analysis for H3K4me3 (Fig. 7A) was carried out by downloading rpm normalized bigwig files of H3K4me3 ChIP-seq data from epigenomesportal [46] for CD38+ B Cells and CD56+ NTK Cells (for both 0–5 years old and 60–65 years old individuals). Heatmaps of H3K4me3 were generated using deepTools2 [47] using the computeMatrix and plotHeatmap function to plot the bigwig signal over genomic regions of cluster 7 as the BED input. The IGV genome browser [48] was used to generate an image of the KCTD1 and IRS2 promoter regions shown in Fig. 7B using downloaded bigwig tracks.

### Analysis environment

Analysis was carried out in a Jupyter [49] analysis environment. Joblib [50], SciPy [51], Matplotlib [52], Seaborn [53], Pandas [54], and TQDM [55] packages were utilized during analysis.

## Results

### Simulated methylation-associated traits

We simulated individuals whose methylation is determined by four factors and their associated epigenetic factors: a uniformly distributed factor approximating age with a non-linear association with methylation status

$$q \sim \mathcal {U}(0,100), s_{Age}=q^{0.5},$$

**Fig. 1:**
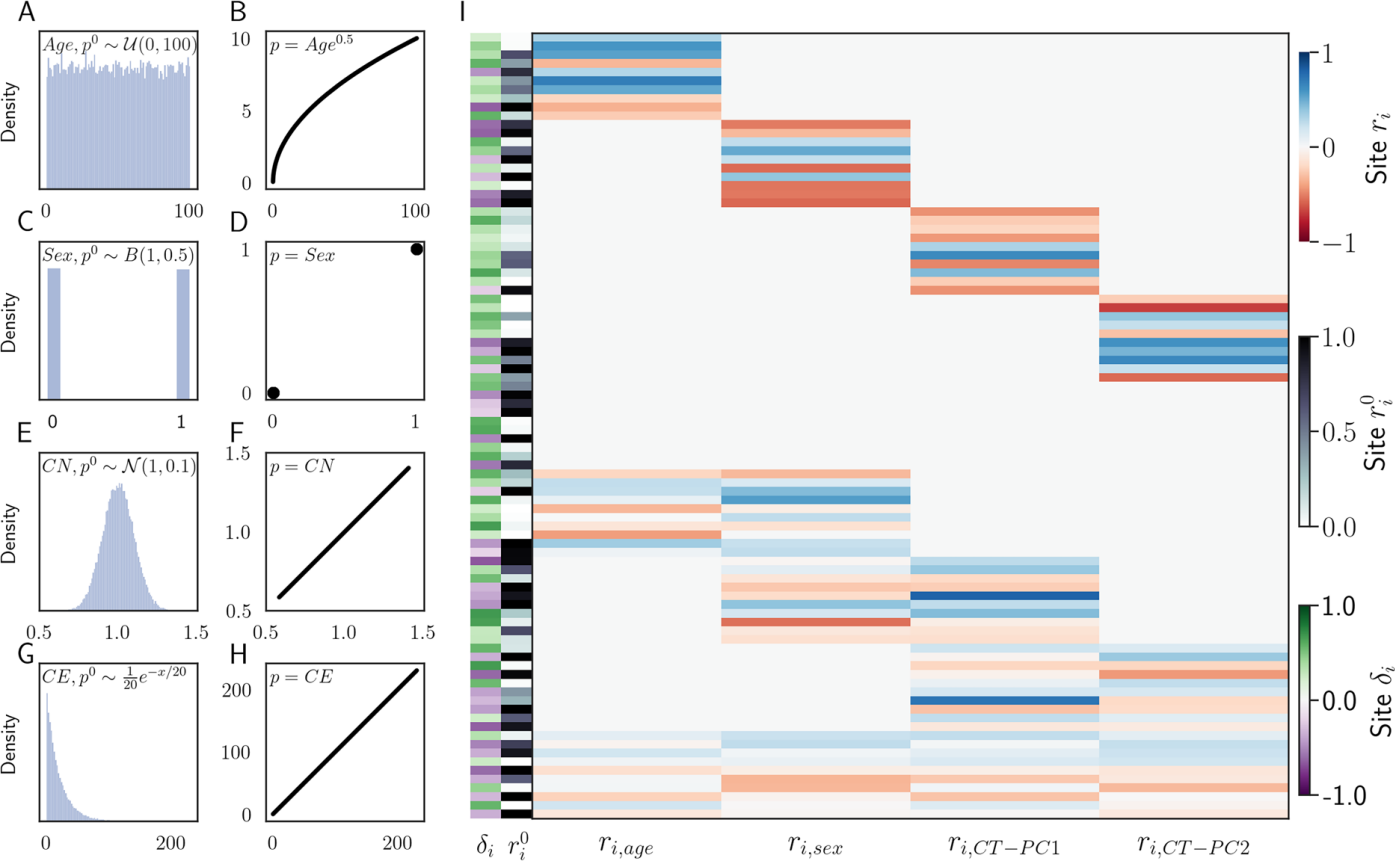
Simulated factors and the association with simulated methylation values. **A** Age with a non-linear association with methylation (**B**). Sex (**C**) with a binary association with methylation (**D**). Normal factor (**E**) with a linear relationship with methylation (**F**). Continuous exponential trait (**G**) with a linear relationship with methylation. **I** Simulated methylation sites. Each simulation site has a starting methylation value $r^0_i$, rate of change associated with each simulated factor $r_{i, factor}$, and range of variation $\delta _i$

Figure 1 A, B a binary distributed trait resembling sex, linearly associated with methylation status

$$q \sim B(1,.5), s_{Sex}=q,$$

Figure 1 C, D a continuous normal (CN) phenotype a linear association with methylation status

$$q \sim \mathcal {N}(1, 0.1), s_{CN}=q,$$

Figure 1 E, F and a continuous exponentially (CE) distributed trait with a linear association with methylation status

$$q \sim \frac{1}{20}e^{-x/20}, s_{CE}=q,$$

Figure 1 G, H.

**Fig. 2:**
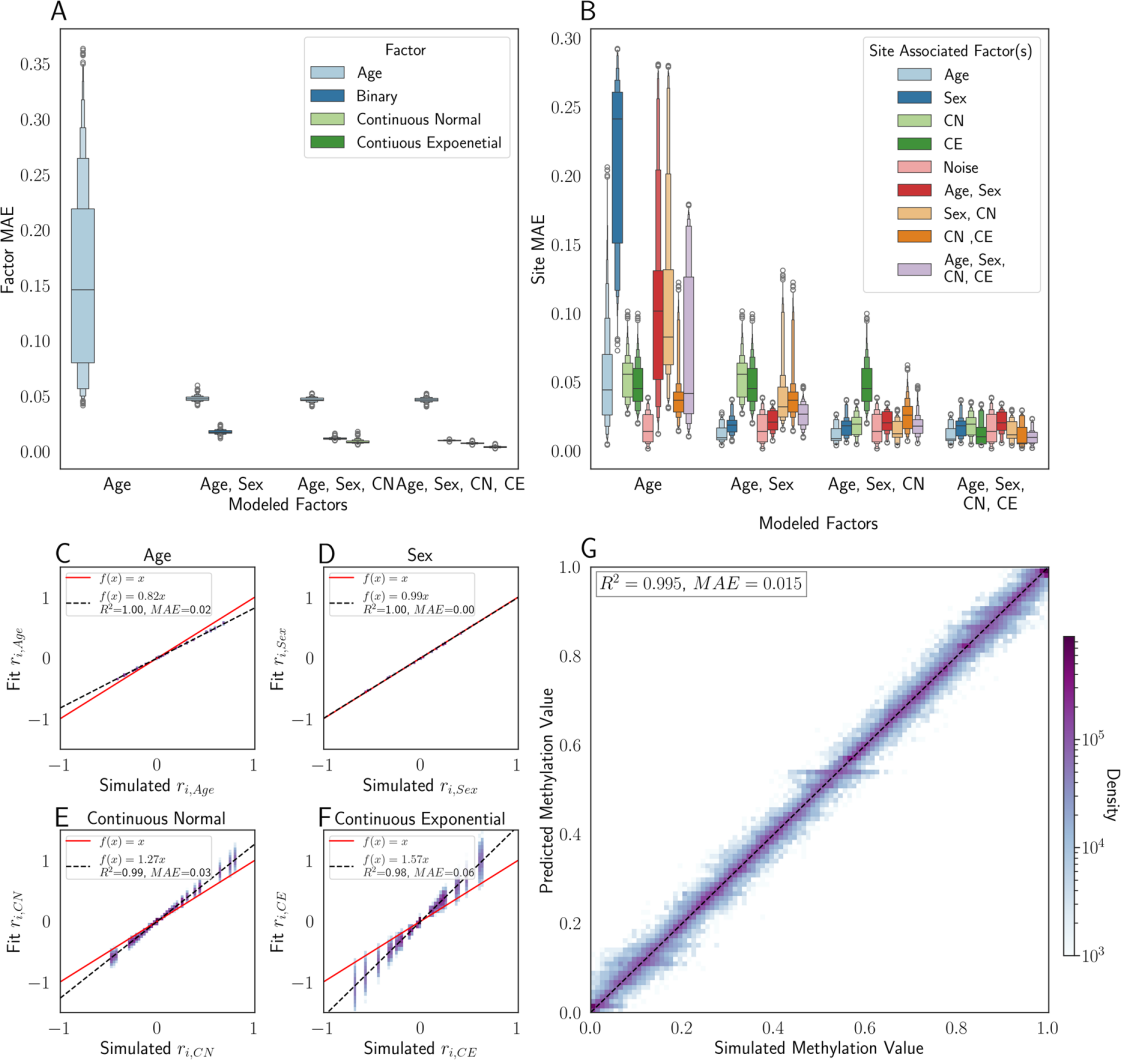
**A** The MAE of the factor predictions on the testing set as multiple factors are modeled simultaneously, and **B** predicted methylation MAE as factors are included in the MSEPM model where the centerline is the 50th quantile and the box with the greatest width contains 50% of the underlying data with each smaller box containing 50% of the remaining data with 6 levels of box width. **C** Model coefficients for age, sex, continuous normal, and continuous exponential factors for models trained $(n = 500)$ with all four simulated factors. **D** Simulated and predicted methylation values for all simulated testing sites across all training fold

We simulated 90 methylation sites (Fig. 1I). We then evaluated the MSEPM model as follows. We simulated 1000 samples with the four epigenetic factors described above. We then simulated methylation values using the simulated site rates. Simulated samples were then split for training ($n=500$) and testing ($n=500$). MSEPM models were then fitted using the values of the input factors, $p^0_{k,j}$. We generated 1000 simulated datasets and fit MSEPM models using four combinations of input factors (Age, Age-Sex, Age-Sex-CN, Age-Sex-CN-CE). Within each simulation, epigenetic state predictions and methylation site predictions were made for all testing samples. All models captured the non-linear association between simulated age and methylation (Supp. Figure 1). As the number of factors in the model is increased, the mean absolute error (MAE) between the predicted epigenetic states and the simulated epigenetic factors decreases (Fig. 2A). Importantly, to accurately assess simulated age, it is necessary to account for the influence of the other simulated factors (Sex, CN, CE).

**Fig. 3:**
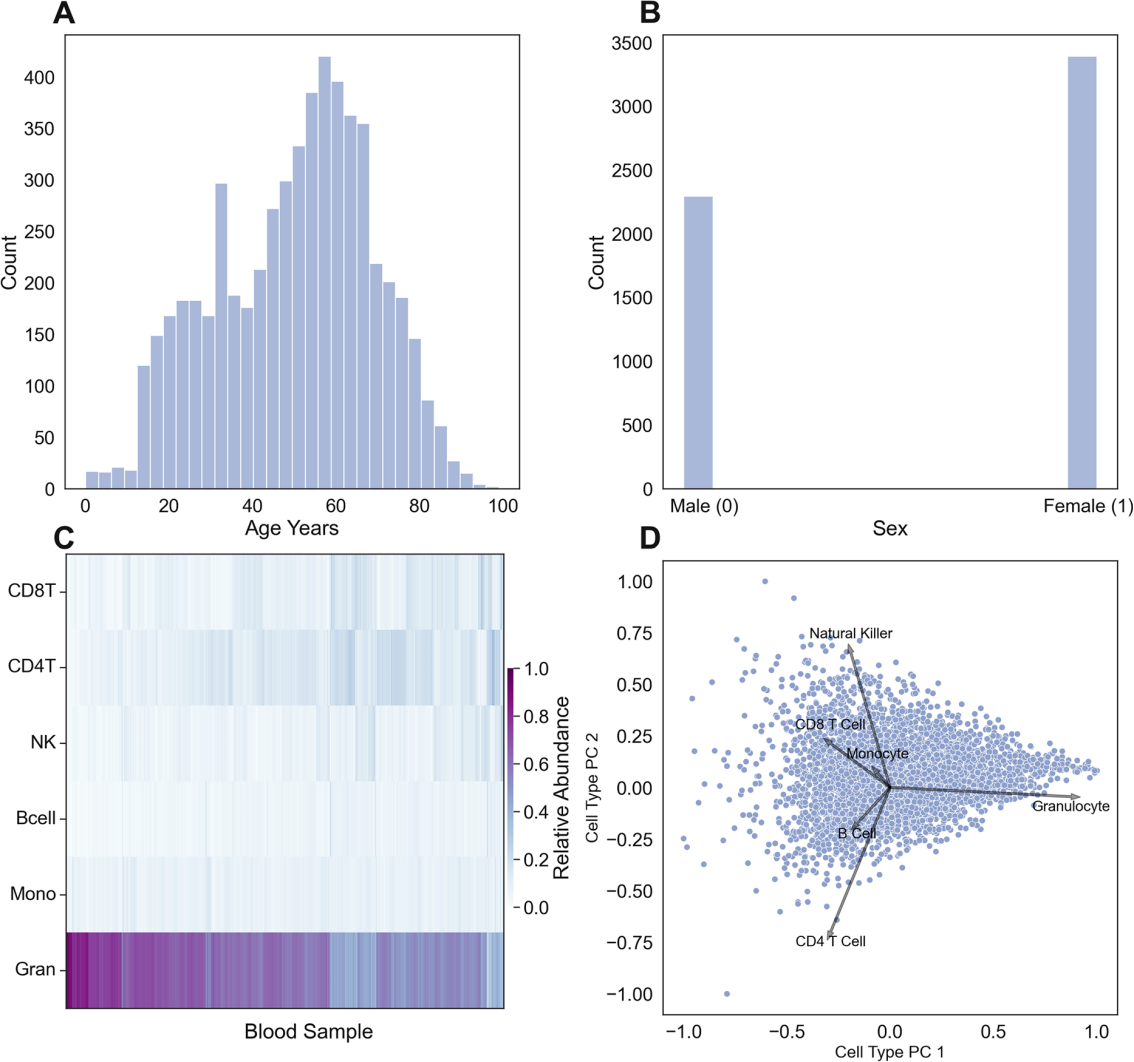
Distribution of age (**A**) and sex (**B**) in aggregate blood dataset. **C** Calculated cell-type composition and **D** loading plot of principal components of cell-type composition in aggregate blood data set

**Fig. 4:**
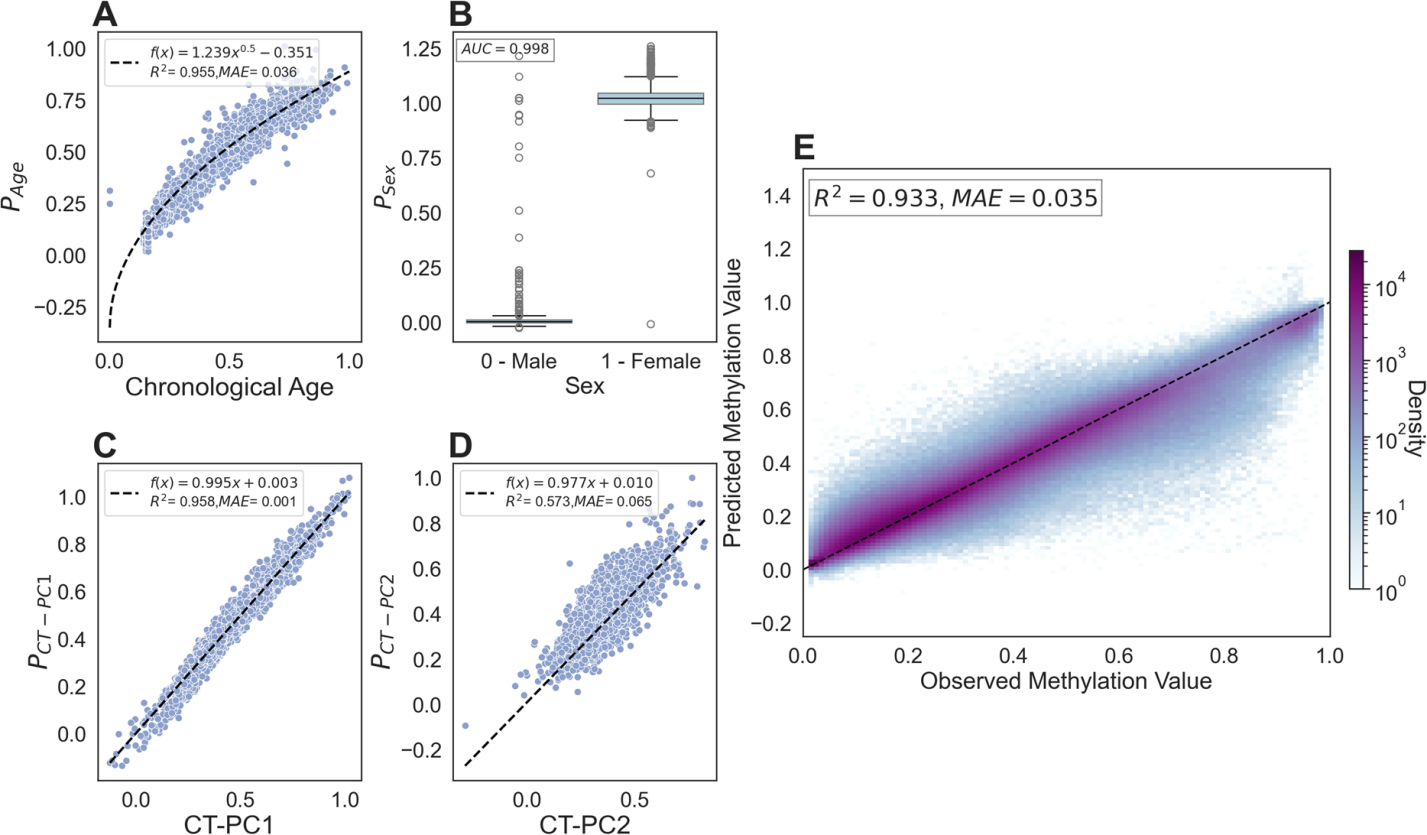
MSEPM model trained with age, sex, CT-PC1 and CT-PC2 predictions within testing set for epigenetic factors **A** age, **B** sex, **C** CT-PC1, and **D** CT-PC2. **E** Observed and predicted methylation values for training set have high concordance $(R^2=0.933, MAE=0.035)$

**Fig. 5:**
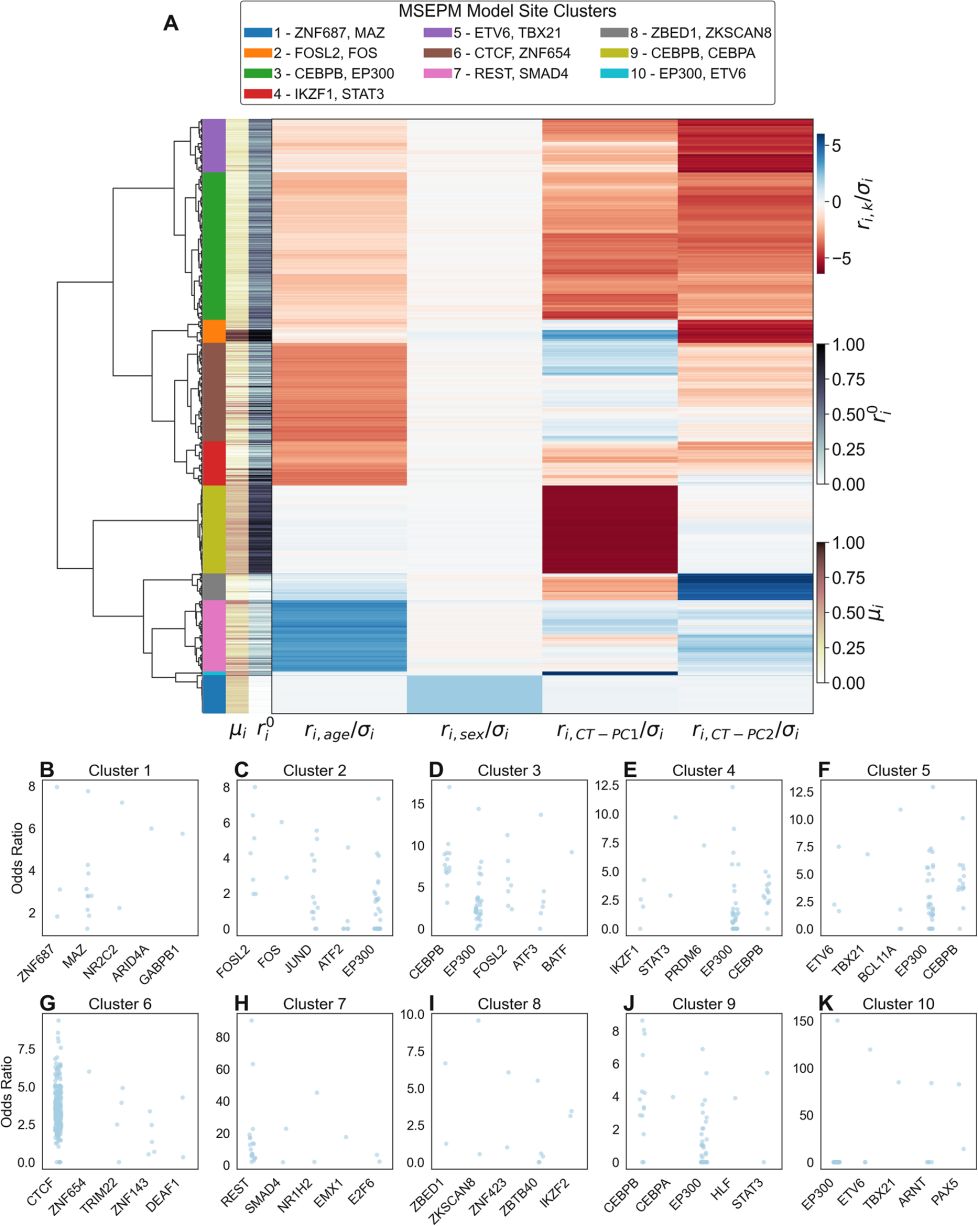
**A** Site clustering by standardized model coefficients. Site clusters show distinct relationships with modeled traits. **B**–**K** Top five enriched transcription factors for clusters 1–10

The MSEPM model generated using all four simulated factors can capture the relative magnitude of the simulated site-specific rates (Fig. 2 C–F). However, the model has difficulty capturing the exact relationship between the simulated factors (age, CN, and CE) and the inferred factors (Fig. 2C, E–F). This is likely due to limitations of the model at capturing non-linear methylation association and a limited training range for normally and exponentially distributed traits. Regardless, the four-factor model can accurately predict the simulated methylation value (Fig. 2D) and site intercept (Supp. Figure 1A). We also assessed the model robustness to variation in the number of samples and sites used for model training by randomly selecting a reduced subset of samples or sites for model training. MSEPM models trained with age, sex, CN, and CE can accurately assess all simulated phenotypes with few samples and sites (Supp. Figure2 B-E).

### Blood MSEPM model

We next applied the MSEPM to real data. We utilized a large aggregated dataset composed of Illumina 450k array data from 17 publicly available datasets [7, 17–32] deposited in the Gene Expression Omnibus[56] (GEO) generated from blood-derived samples (whole blood, peripheral blood lymphocytes, and peripheral blood mononuclear cells). The aggregate data spanned a wide age range (0.0–99.0 years, Fig. 3A), contained more predicted females ($n=3392$) than males ($n=2295$, Fig. 3B), and reasonably predicted cell-type abundance estimates (Fig. 3C). The first principal component of a PCA model trained cell-type abundance estimates (CT-PC1) is largely driven by the relative abundance of granulocytes (Fig. 3D), while the second PC (CT-PC2) captures relative differences in the abundance of differentiated lymphocytes (Fig. 3D).

We trained MSEPM models using methylation sites ($n=778$) that were correlated with the observable input factors. MSEPM models were fit using four combinations of input factors (Age, Age Sex, Age Sex CT-PC1, and Age Sex CT-PC1 CT-PC2). The association between the fit epigenetic factor predictions against the input modeled factors was assessed by fitting a trendline between epigenetic state predictions and scaled continuous input factors using the state prediction made for the MSEPM model trained with all four input factors. The performance of the MSEPM model was then evaluated using the testing samples ($n=4,082$). The performance of the MSEPM largely closely resembles the simulation results. All four MSEPM models capture the non-linear relationship between age and methylation status (Supp. Figure 6). The epigenetic state prediction associated with age improves as the underlying methylation data are more fully explained through the addition of epigenetic factors (Supp. Figure 6). The MSEPM model fit with age, sex, CT-PC1, and CT-PC2 can accurately model the associated epigenetic state for each factor (Fig. 4 A–D) and accurately predicts the methylation levels at individual sites ($R^2=0.935$, $MAE=0.035$, Fig. 4E). The trained MSEPM produces a collection of methylation site models that can help explain the association between modeled factors and methylation status.

### Analysis of chromatin regulators of site clusters

We evaluated the relationship between sites that are influenced by age, sex, CT-PC1 or CT-PC2, and potential regulatory factors by performing overlap enrichment analysis of these sites with transcription factor chromatin immunoprecipitation peaks present in the ENCODE V4[38, 39] release. We first identified sites with similar coefficients of epigenetic factors through hierarchical clustering. The resulting tree was cut at a height of 18 to produce ten distinct clusters with clear associations to the modeled factors (Fig. 5A).

The site clusters largely conform to underlying biological expectations. Cluster one contains sites that are wholly associated with sex status and localized to the X chromosome (Supp. Table 1) and is enriched for peaks of transcription factors associated with sex-specific regulation such as MAZ [57]. Clusters nine and ten contain sites whose methylation status is largely driven by CT-PC1 and are enriched for transcription factors associated with granulocyte development (CEBPB, CEBPA, EP300, ETV6) [58, 59]. Similarly, clusters two, five, and eight are associated with CT-PC2 and are enriched for transcription factor peaks associated with immune development (ZBED1, ETV6, FOSL2, FOS, TBX21). Clusters four and six are associated with loss of methylation with age. Cluster six is highly enriched for CTCF binding sites; CTCF is known to increase at sites where methylation is lost during aging [60]. Cluster four is enriched for STAT3 whose activation during exercise is age-dependent [61, 62]. Cluster seven is associated with the accumulation of methylation with age and is enriched for immunoprecipitation peaks for aging associated transcription factors SMAD4 and RE1-Silencing Transcription Factor (REST). SMAD4 encodes a protein involved in the transforming growth factor beta (TGF-$\beta$) signaling pathway. Age-related dysregulation of TGF-$\beta$ has been linked to reduced skeletal muscle regeneration [63, 64], and SMAD4 polymorphisms are associated with longevity [65]. REST is a transcriptional repressor of neuron-specific genes in non-neuronal cells [66, 67]. REST expression is upregulated in aged prefrontal cortex tissue, and the absence of REST expression is associated with cognitive impairment [68] and cellular senescence in neurons [69].

### Analysis of sites with age-associated increases in methylation

**Fig. 6:**
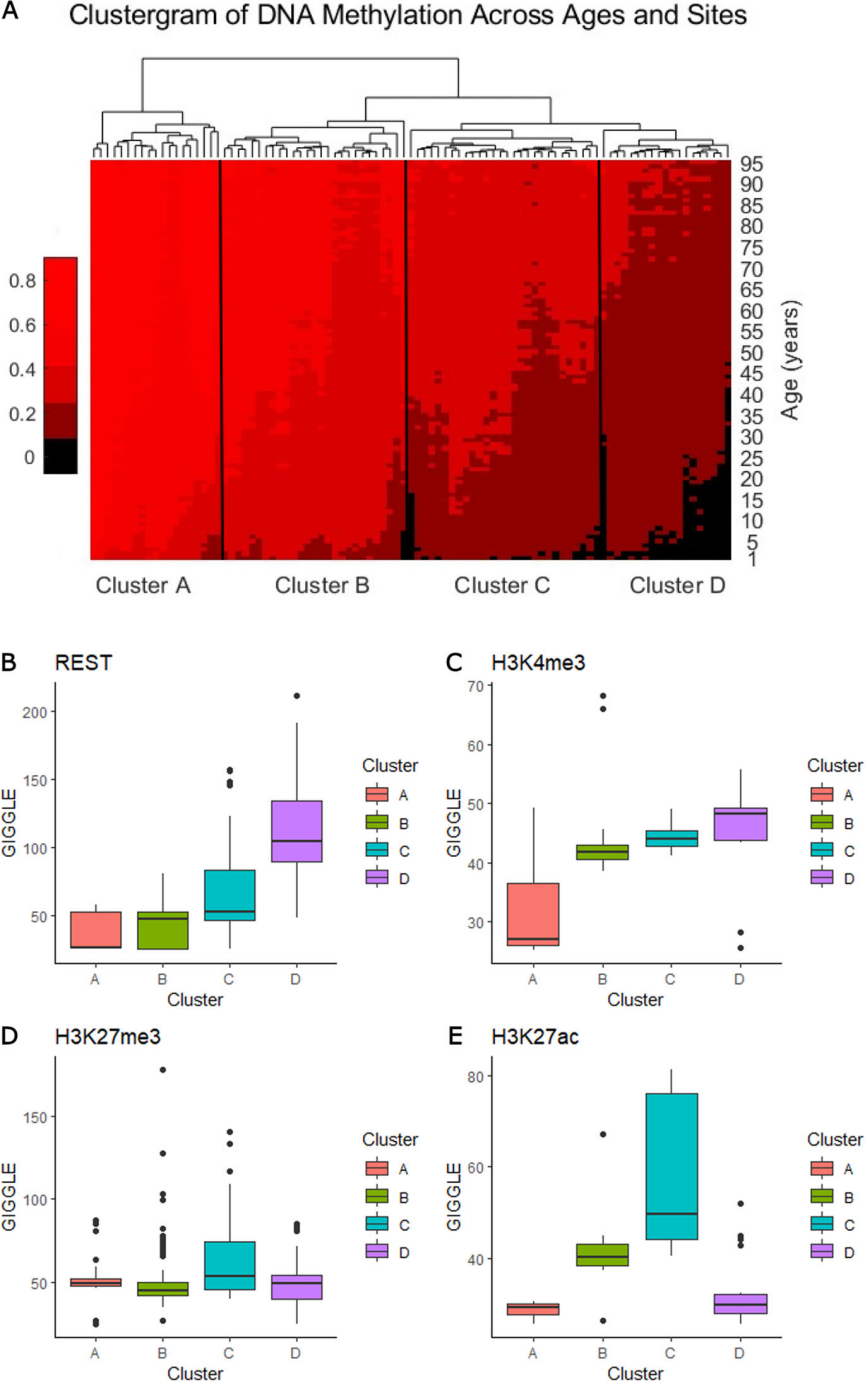
**A** Heatmap of H3K4me3 ChIP-seq enrichment for two different blood cell types (CD38^+^ B Cells and CD56^+^ NTK Cells) in two cohorts of individual within 0 to 5 years old and 60 to 65 years old. The average level within 2 kb up and downstream for centered genomic regions of cluster 7 is represented above the heatmap. **B** Genome browser view of H3K4me3 levels in each cohort at the promoter regions of *KCTD1* and *IRS2* genes

Because of our interest in the mechanisms that underlie ages associated increase in methylation, we focused on cluster seven, as these sites have methylation increases that depend primarily on age rather than sex and cell types. Cluster 7 consisted of 93 CpG sites. To obtain an independent measure of how these sites change with age, we obtained age-associated methylation

data from the EWAS Data Hub [70], with a focus on whole blood methylation. The dataset consisted of about 1600 individuals with ages ranging from 0 to 113 years old [71]. We clustered the sites based on age-associated methylation levels, meaning the rate of methylation was based on age for each marker. Each site was organized into an ordered matrix with methylation levels at each age and then grouped into four sub-clusters: A, B, C, and D. As seen in Fig. 6A, cluster A had the highest average methylation across ages, and each consecutive cluster had a decrease in average methylation. We next examined chromatin accessibility, transcription factors, histone marks, and genes associated with each cluster. As shown in Supp. Figure 7, genes proximal to cluster 7 sites were lowly expressed in blood compared to other tissues. We analyzed chromatin accessibility, transcription factors, and histone marks associated with these four groups. We computed levels of H3K27ac, H3K27me3, H3K4me3, and H3K9me3 across the four sub-clusters. As seen in Fig. 6C, H3K4me3 increased from clusters A through D. Figure 6E shows that H3K27ac increased from clusters A through C, but then decreased in D. These results suggest that sub-cluster D is enriched for bivalent domains, characterized by H3K4me3 and H3K27me3.

**Fig. 7:**
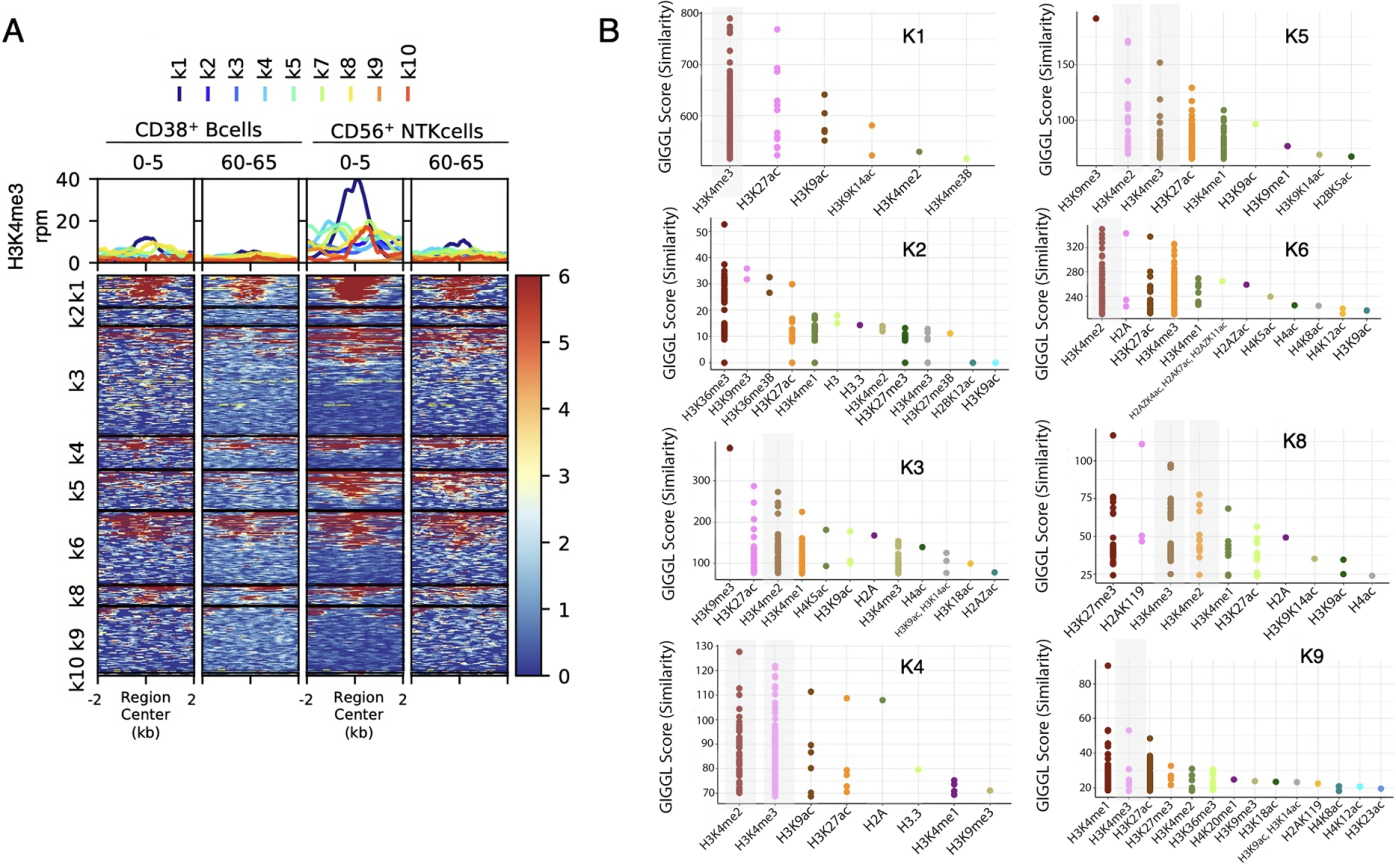
**A** Heatmap of H3K4me3 ChIP-seq enrichment for two different blood cell types (CD38^+^ B Cells and CD56^+^ NTK Cells) in two cohorts of individual within 0 to 5 years old and 60 to 65 years old. The average level within 2 kb up and downstream for centered genomic regions of cluster 7 is represented above the heatmap. **B** Genome browser view of H3K4me3 levels in each cohort at the promoter regions of *KCTD1* and *IRS2* genes

Based on these results, we hypothesize that the mechanisms that underlie the gain of methylation with age at these bivalent promoters are the age-associated loss of H3K4me3. It is well established that the presence of trimethylation on H3K4 inhibits de novo methylation, and this effect explains the hypomethylation that is typical of promoters, including bivalent promoters. We therefore hypothesize that the gain of methylation at these sites may be caused by an age-associated loss of H3K4me3. In order to demonstrate that H3K4me3 decreases with age for genomic regions where DNA methylation increases, we used published H3K4me3 ChIP-seq data from epigenomesportal [46]. We selected two different blood cell types, CD38+ B Cells and CD56+ NTK Cells, and plotted the H3K4me3 signal of young (0 to 5 years old) versus old individuals (60 to 65 years old) over genomic regions of cluster 7 (Fig. 7A). Our analysis shows that younger individuals have higher levels of H3K4me3 compared to older ones (Fig. 7A) as also shown for two selected genomic loci of cluster 7 (the promoters of KCTD1 and IRS2 genes) where we can observe a marked decrease in the levels of H3K4me3 as age increases (Fig. 7B). Altogether, these data suggest that genomic regions whose DNA methylation is increased with age exhibit an age-dependent loss of H3K4me3, thus showing an inverse correlation between DNA methylation and H3K4me3 at these genomic loci.

## Discussion

Epigenetic clocks are widely used tools to study human aging and health. Despite their widespread use, the biological interpretability of the models is limited. A methylome is influenced by many different biological processes occurring simultaneously over time that may differ among individuals. Epigenetic clocks, while producing accurate predictions of age, attempt to capture this complexity through a single dependent variable. Additionally, the penalized regression-based methods used to fit most epigenetic clocks select sites that minimize, or regress out, the influence of other factors and omit groups of sites that are correlated. To overcome these limitations, here, we propose a multidimensional extension of the EPM model, the MSEPM.

In contrast to previous methods, the MSEPM aims to simultaneously model the effect of multiple factors on the methylome. The simulation and blood MSEPM models show that concurrently modeling age, cell-type composition, and sex can minimize model residuals when compared with the MSEPM model fit with age only. The residual of the age only model is often interpreted as a measure of age acceleration. When multiple methylome-associated traits are modeled simultaneously, this residual can be explained directly by other factors and the association between the methylome and a trait of interest can be inferred.

Additionally, the individual methylation site linear models fit as part of the MSEPM optimization can provide information about the relationship between modeled factors and site-specific biology. To this end, we find that the blood MSEPM model conforms to expected biology. Sites with a strong sex association localize to the X chromosome and sites associated with cell types are enriched for transcription factors associated with the development of immune cells.

CpG sites that are primarily affected only by age in the blood MSEPM model are of particular interest. As others have previously described, sites that progressively lose methylation over time are strongly enriched for CTCF [72, 73]. As CTCF plays a key role in long-range chromatin interactions, this may suggest that there are age-associated changes in three-dimensional chromatin structure and that the structure may become more disordered with age. In fact, alterations in CTCF binding and function with age have been implicated in the pathogenesis of various age-related diseases, including cancer. For example, changes in the chromatin structure and gene expression due to altered CTCF binding can contribute to the genomic instability and altered cell proliferation characteristic of cancerous cells (Hnisz et al., 2016; Phillips et al., 2009).

We identified a cluster of sites that showed increasing methylation with age and that were not significantly affected by other factors. We found that these sites are enriched for the transcription factor REST. The RE1-Silencing Transcription Factor (REST), also known as Neuron-Restrictive Silencer Factor (NRSF), is a key regulatory protein involved in the development and differentiation of neurons. It plays a crucial role in neurogenesis, in neuronal differentiation, and in the maintenance of the neuronal phenotype by regulating gene expression [74]. REST achieves this by binding to the neuron-restrictive silencer element (NRSE) or RE1 sites in the DNA, leading to the repression of gene transcription in non-neuronal cells or in neuronal progenitor cells, ensuring that neuronal genes are expressed only in neurons [66, 75, 76]. The fact that this factor is enriched at the positively age-associated sites suggests that these sites are likely expressed in neuronal cells but not in blood. In fact, this is what we find when we examine the tissue-specific expression of the genes proximal to these sites.

We also examined the histone modifications associated with the positively age-associated sites and found that they were enriched for H3K4me3 and H3K27me3. These sites are characteristic of bivalent promoters. Bivalent promoters play a crucial role in the regulation of gene expression during development and differentiation. Characterized by the simultaneous presence of both activating (H3K4me3) and repressive (H3K27me3) histone modifications, bivalent promoters mark genes that are poised for transcription but are not actively transcribed. This dual modification serves as a regulatory mechanism, ensuring that genes essential for differentiation and development are ready to be activated at the appropriate time. Bivalent domains are predominantly found in embryonic stem cells and are crucial for maintaining the cells in a pluripotent state, allowing for the rapid activation or repression of gene expression in response to developmental cues. The significance of bivalent promoters extends to their role in cell fate decisions, where they contribute to the tight control of developmental pathways and the maintenance of stem cell identity [77, 78]. Our results suggest that the bivalent promoters we identified in blood are inactive (as seen by the fact that the proximal genes are not expressed). However, the fact that DNA methylation at these sites increases with age suggests that they may be losing H3K4me3 with age. H3K4me3 is a critical regulator of DNA methylation as it inhibits the binding of DNMT3 to histones, as the DNMT3 ADD domain preferentially binds to the unmethylated H3K4 residue [79]. This explains why promoters, which are enriched for H3K4me3, are generally hypomethylated. Our results suggest that there must therefore be an age-associated loss of H3K4me3 at these bivalent promoters. That is in fact what we saw when we examined these marks in B cells and Nk cells of both young and old individuals. These mechanisms further suggest that the age associated DNA methylation increases may not have a functional consequence in blood and that their proximal genes remain repressed throughout life.

In conclusion, we introduced a multidimensional extension of the Epigenetic Pacemaker, the MSEPM. The MSEPM is capable of accurately modeling multiple methylation-associated factors simultaneously. This paradigm can elucidate the site-specific regulation underpinning methylome dynamics. It allows us to characterize the mechanisms underlying age-associated increases in methylation sites, suggesting that these were caused by the loss of H3K4Me3 at bivalent promoters of genes that are silenced in blood. The MSEPM is available under the MIT license at https://github.com/NuttyLogic/MultistateEpigeneticPacemaker.

### Supplementary information

All analysis code, data processing code, and supplementary material associated with this manuscript can be found at https://github.com/NuttyLogic/MSEPMManuscript. The methylation simulation utility can be found at https://github.com/NuttyLogic/MethSim. The data supporting these findings are openly available at GEO under the series GSE87640, GSE87648, GSE51057, GSE51032, GSE87571, GSE125105, GSE42861, GSE69138, GSE111629, GSE128235, GSE121633, GSE73103, GSE61496, GSE59065, GSE97362, GSE156994, GSE128064, and GSE43976.

## Supplementary Information

Below is the link to the electronic supplementary material.

Supplementary file 1 (pdf 8546 KB)

## Data Availability

The data supporting our findings are available at GEO under the series GSE87640, GSE87648, GSE51057, GSE51032, GSE87571, GSE125105, GSE 42861, GSE69138, GSE111629, GSE128235, GSE121633, GSE73103, GSE61496, GSE59065, GSE97362, GSE156994, GSE128064, and GSE43976.
