# Supplementary material for: The Multi-State Epigenetic Pacemaker enables the identification of combinations of factors that influence DNA methylation
Source: GeroScience. 2024 Nov 16;47(2):2439–54. doi: 10.1007/s11357-024-01414-7 (PMC11979089; doi:10.1007/s11357-024-01414-7)
Supplement: Supplementary file 1 — (pdf 8546 KB) [file 11357_2024_1414_MOESM1_ESM.pdf]

The Multi-State Epigenetic Pacemaker enables the identification of combinations of factors that influence DNA methylation - Supplementary Information

Colin Farrell[1,4], Keshiv Tandon 1, Roberto Ferrari[2], Kalsuda Lapborisuth[1], Sagi Snir[3], and Matteo Pellegrini[1,4]

[1]Dept. of Molecular, Cell and Developmental Biology;
University of California, Los Angeles, CA 90095, USA;;
[2]Dept. of Chemistry, Life Sciences and Environmental Sustainability, Laboratory of Molecular Cell Biology of the Epigenome (MCBE), University of Parma, Italy;
[3]Dept. of Evolutionary Biology, University of Haifa, Israel;
[4]Corresponding Authors; colinpfarrell@gmail.com, matteop@mcdb.ucla.edu

1 Supplementary Table Descriptions

Supplemental Table 1: MSEPM model parameters for MSEPM blood model trained against age, sex, CT-PC1 and CT-PC2.
Supplemental Table 2: Sample characteristics for GEO samples used in MSEPM blood model training, validation, and testing.
Supplemental Table 3: LOLA transcription factor binding results.
Supplemental Table 4: Simulated methylation site parameters.

2 Supplementary Figures

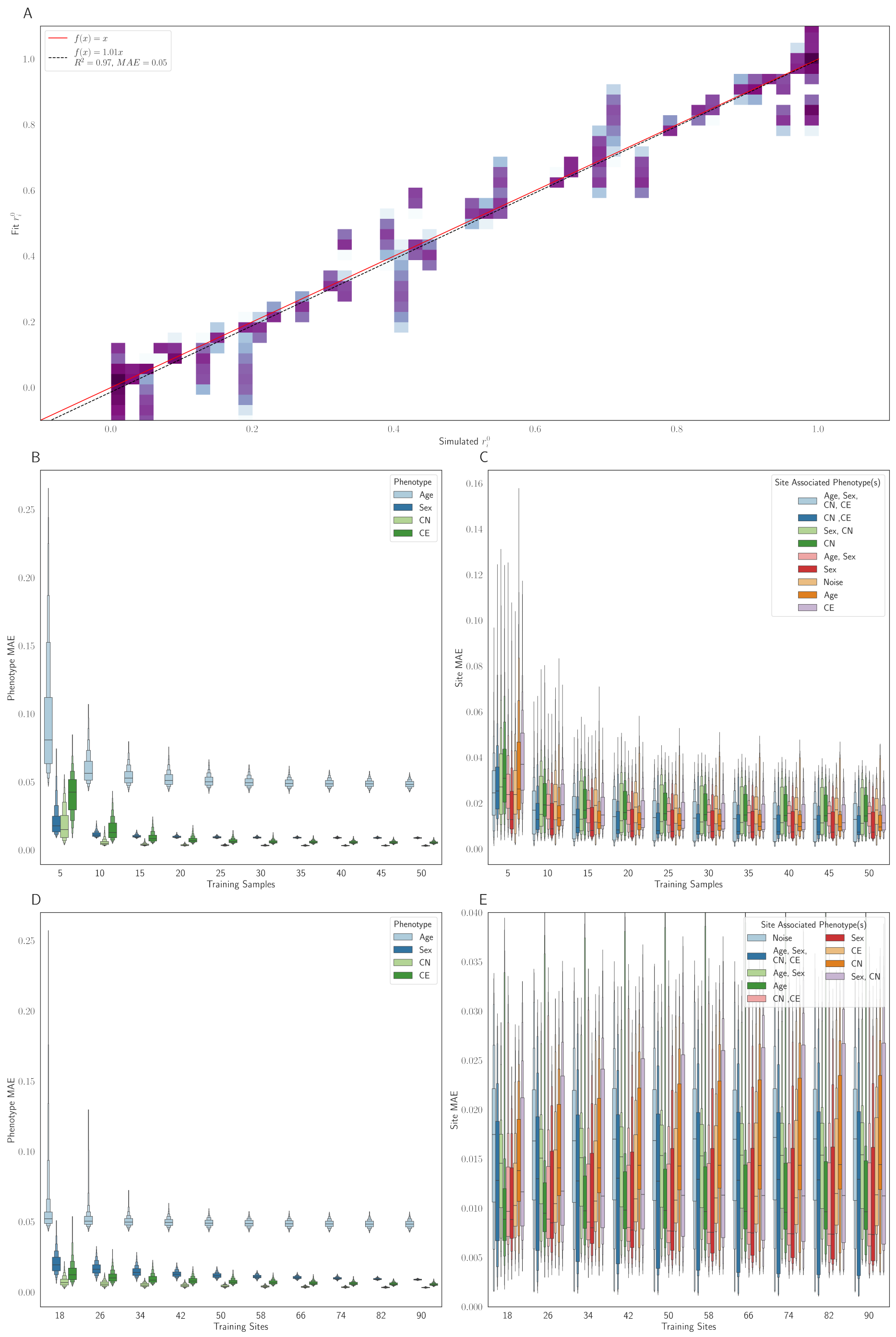

Supp. Figure 1: Simulated methylation site intercept accurately modeled with MSEPM four factor model (A). Simulation phenotype (B) and site methylation (C) prediction MAE for four-factor MSEPM models fit with a varying number of training samples and sites (D-E).

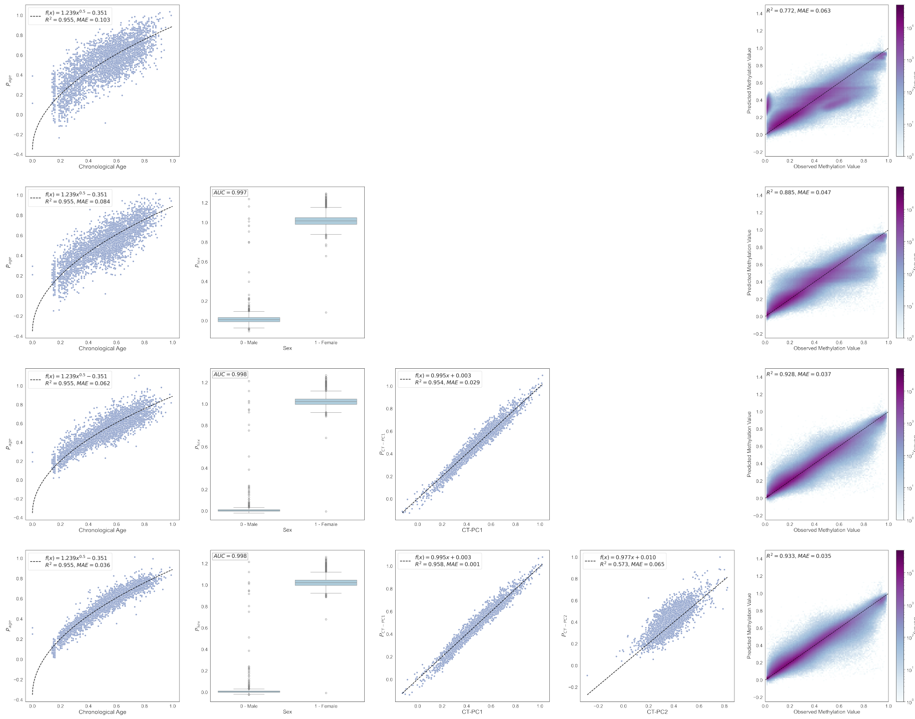

Supp. Figure 2: MSEPM testing blood model predictions for MSEPM model fit with only age (first row), age / sex (second row), age / sex / cell type PC1 (third row), and age / sex / cell type PC1 / cell type PC2 (fourth row).

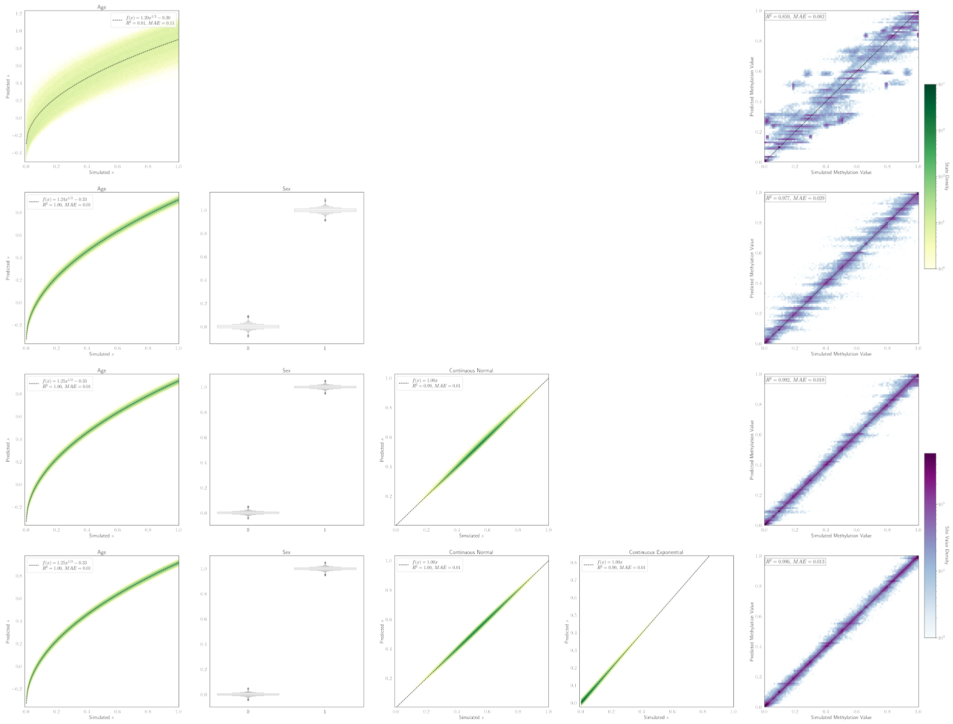

Supp. Figure 3: Simulated testing model predictions for MSEPM model fit with only age (first row), age / sex (second row), age / sex / CN (third row), and age / sex / cell type PC1 / cell type PC2 (fourth row).

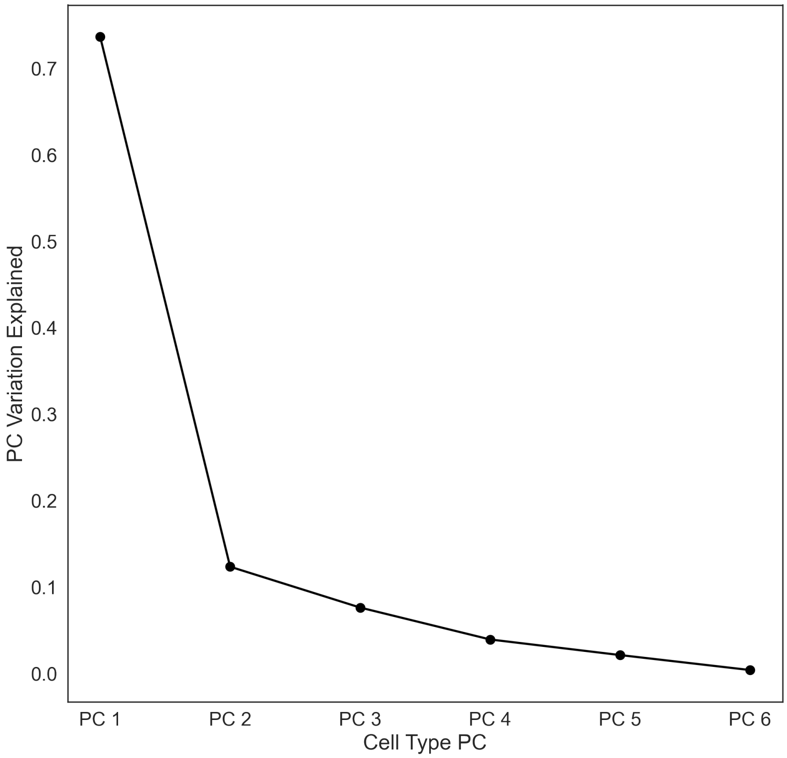

Supp. Figure 4: Cell type principal component analysis scree plot.

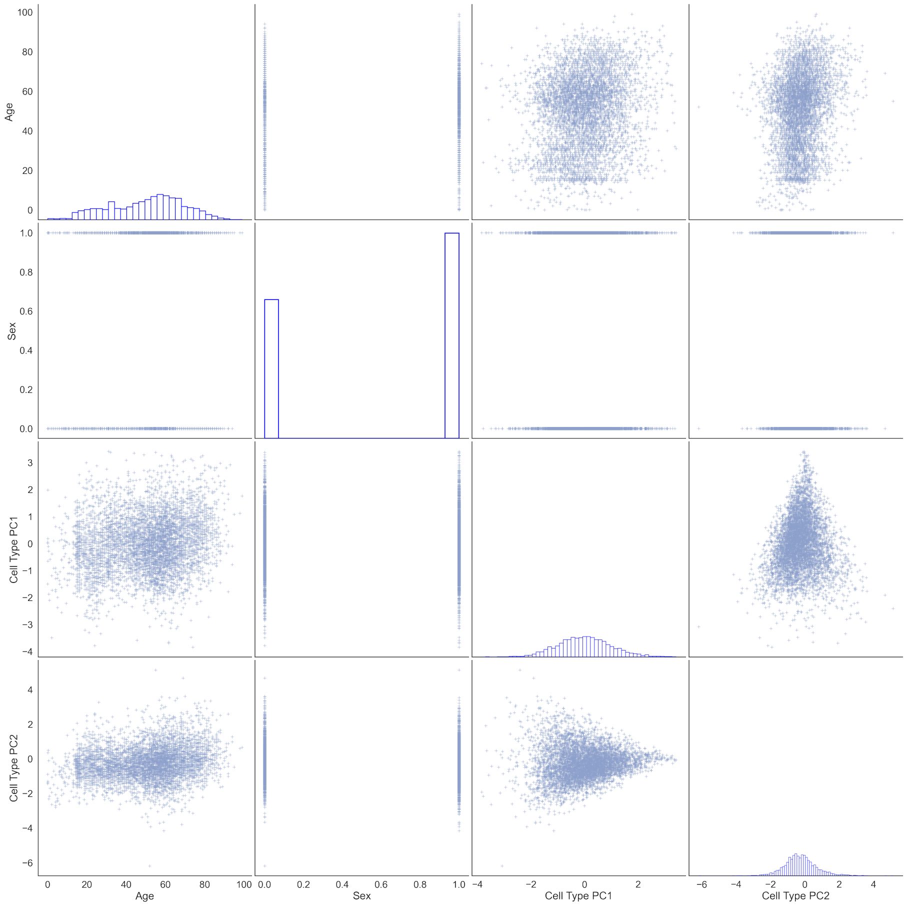

Supp. Figure 5: Pairwise bivariate distributions, and single factor distributions plots, for GEO factor data (age, sex, CT PC1 and CT PC2) used in MSEPM model training.

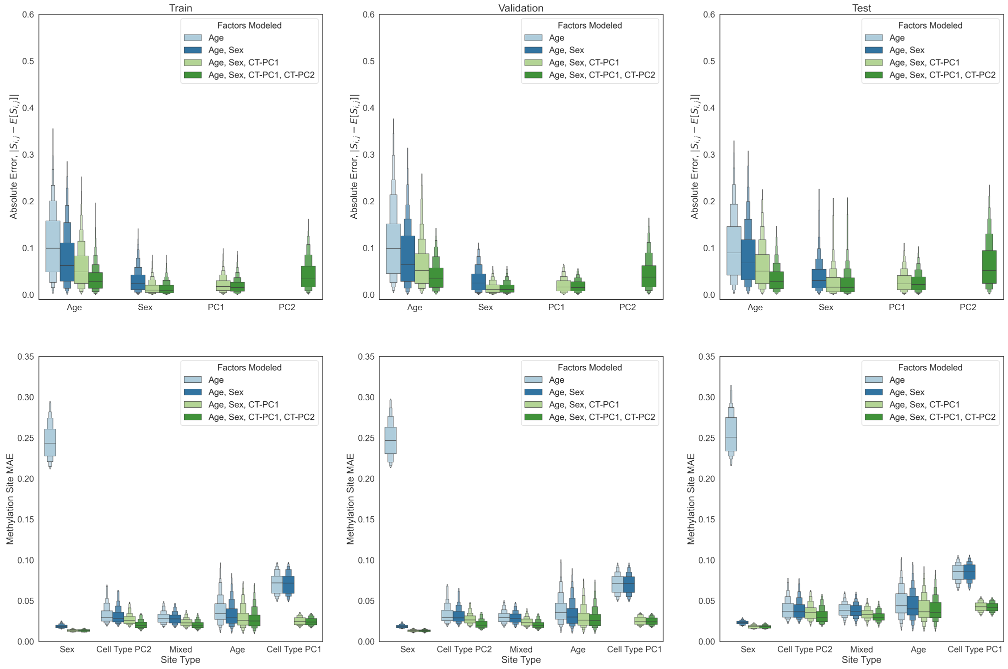

Supp. Figure 6: Methylation site and sample factor prediction error for models trained with 1 to 4 factors for training, validation and testing sets.

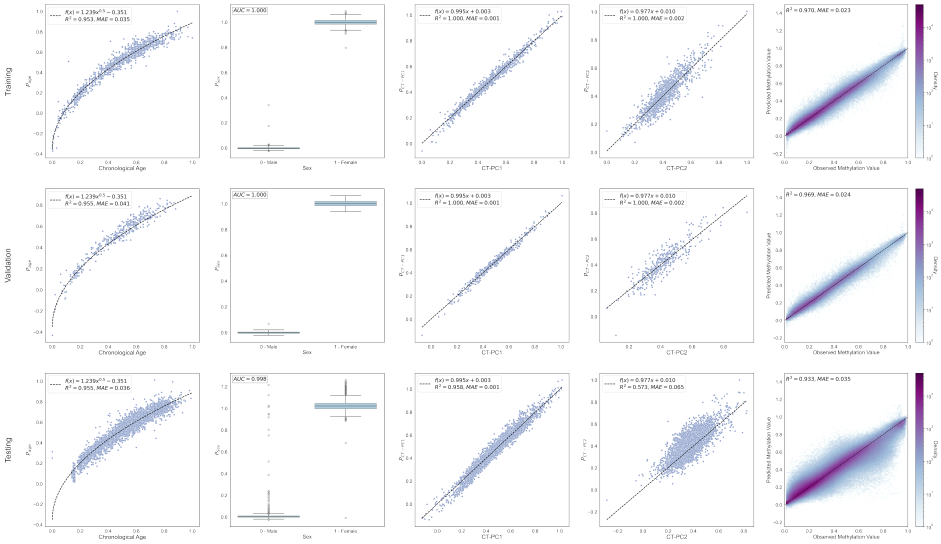

Supp. Figure 7: MSEPM blood model predictions for MSEPM model fit against age, sex, CT PC1 and CT PC2 for training, validation and testing sets.
